# Unbending the Light: Changing Laws and Policies to Make Transgender Health Visible; Reflections of an Advocate

**DOI:** 10.1017/jme.2022.90

**Published:** 2022

**Authors:** Jamison Green

**Affiliations:** 1. INDEPENDENT SCHOLAR

**Keywords:** Transgender Healthcare, Health insurance, WPATH, Standards of Care, HBIGDA

## Abstract

This essay describes an instrumental advocate’s development, engagement, and accomplishments in transgender health at the intersection of law and medicine. Reflecting on the evolution of insurance policy reforms in conjunction with the need to increase the availability of clinicians who can understand and respectfully treat transgender patients, the author demonstrates how visibility, tenacity, and ingenuity can create far-reaching change.

There is something twisted about the way society has historically viewed transgender people, people who are gender incongruent, gender diverse, gender queer. We’re seen through prisms of ingrained beliefs about the human condition, most of which are already distortions of facts. Each of us must learn to find ourselves, then to accept ourselves, and then to find our place in the world. It should not be surprising that trans people, who don’t follow an easy path, might be regarded as inconvenient at best and downright disgusting at worst.

I struggled for about 18 years to come to terms with the fact that I was not like the other girls or the other boys, and when I would wear “girls’ clothes,” people would ask me, “Are you a boy or a girl?” And when I wore “boys’ clothes,” people would either not notice me, or they would tell me I was “cute.” By the time I was in college, I had come up with a word for my situation: I was “cross-gendered.” By that I meant that I felt like there was a wire crossed between my brain and my body. There was nothing wrong with me as a person, as a student, as a friend to anyone, or as a member of my family. Except that now that I was 18 years old, I vowed I would never wear “girls’ clothes” again, even if it made my family upset, which it did.

It took roughly another 18 years before I became fully cognizant that it was possible to undergo a medical transition from female to male. By that time, I had earned a Master of Fine Arts degree in writing, worked for 3 years as the first woman construction cable splicer for Pacific Northwest Bell Telephone Company, then, among other odd jobs, had taught legal writing in a special summer institute program for women and minorities who had been conditionally admitted to a private university law school, and worked my way up from unemployed to temporary warehouse worker, to medical writer, to manager of technical publications at a series of computer design and manufacturing companies. By the time I was 38 years old, I was vice president of operations at a publicly-held software publishing company, and people were still uncertain what sex I was.

In my capacity as a medical writer, in 1980, I had my first successful policy breakthrough over a surgical procedure that was deemed “experimental” and therefore would not be covered by insurance. It was arthroscopic knee surgery: I had developed a bucket-handled tear of the meniscus, and my boss, the VP of R&D at my company, recommended that I see the surgeon who was the Chief of the College of Arthroscopic Surgery, assuming he would take me as a patient. He did, and because he was a friend of my boss, he agreed to accept $25 per month from me as payment for his work, since that was all I could afford (as far as I could foresee, this would be my monthly payment for the rest of my life). The surgery was successful, and after dutifully making my meager payment for several months, it dawned on me that I could write a letter to the insurance company to explain the benefits of arthroscopic surgery over the conventional large incision technique that they would have paid for without question. In my letter, I described my experience (not even an aspirin for post-op pain, only a few days of recovery time) and compared it to the months-long and very painful recovery, risk of infection, lost work, lost income, etc., experienced by those who underwent the procedure insurance would approve because they could not otherwise afford the necessary surgery. I proposed that removing arthroscopic procedures from the “excluded” list due to the “experimental nature of the technique” should be reconsidered due to its clear cost effectiveness and benefit to patients. I also sent a copy to my surgeon. A few months later, I was amazed to hear from the surgeon that the insurance company had paid him an amount equal to what I owed him, minus $300.00, and he offered me $300.00 in exchange for the use of my letter to help other patients. I was thrilled to accept his offer. It wasn’t much longer before arthroscopy was a covered procedure in health benefit policies across the country because my surgeon had the leverage to influence that change. But it wasn’t lost on me that the ability to construct a concise argument supported by evidence and deliver it to the right place was also necessary to make policy changes.

By the time I was in college, I had come up with a word for my situation: I was “cross-gendered.” By that I meant that I felt like there was a wire crossed between my brain and my body. There was nothing wrong with me as a person, as a student, as a friend to anyone, or as a member of my family. Except that now that I was 18 years old, I vowed I would never wear “girls’ clothes” again, even if it made my family upset, which it did.

My partner and I were on the leading edge of the “lesbian baby boom” of the early 1980s; she gave birth to our children, a girl in 1985, and a boy in 1989, full siblings courtesy of the Sperm Bank of Northern California. It was during her gestation of our son (we did not know the sex of the fetus) that I struggled most deeply with gender dysphoria. Realizing, fearfully, that I needed to change my sex, or I would remain an “in-between” forever because I was incapable of being a woman, I joined a newly formed information and support group in San Francisco, called FTM, where I met a few dozen trans men at various stages of medical transition. The group met quarterly, and (reminder…) the internet was not well developed or available to most Americans, so real information was sparse. But I did my internal homework, and, with my partner’s blessing, I applied to the nation’s then most prestigious sex reassignment program, known as the Sex Reassignment Program at Stanford. After taking the MMPI^1^ test, having a few evaluation meetings, and waiting about four months, I was accepted as a candidate and began my medical transition with testosterone in the fall of 1988. I looked forward to chest reconstruction surgery the following spring and anticipated having genital reconstruction a year or so later. Not much would change for me, I reasoned. I would just be more comfortable in my body. I would have my sex changed, and then go home and mow my lawn, no big deal.

Just a few days after our son was born, I realized my partner was avoiding me, but she refused to admit it or to speak with me at length about anything other than logistics concerning our children. With my paternity leave over, I went back to work. About two months later, I had my “top surgery,” and shortly after that my partner announced that our relationship was over, and she was leaving. I had no choice but to sell our modest home and split the profits with her to help support her, and of course I would pay child support. It was a difficult time emotionally for me — I was heartbroken, frankly — and I listened more closely to the stories of the other men at the FTM support group meetings, feeling more connection with them than I had before. Even when our experiences had little in common on the surface, there were fears and concerns and problems and joys we all shared categorically, especially access to basic health care and transition-related treatments.

Insurance will never pay for this, both clinicians and program administrators told us. I began to think about why that might be, and what might be done about that barrier. Knowing the considerations would likely be different from arthroscopy, even though both were classified as “experimental,” I reasoned that consumers alone would have little leverage with insurance companies when it came to transgender care. We would need bigger guns, or probably brighter flashlights. We needed people with the leverage to implement policy change to see that our need was real.

In 1992, I was invited by the San Francisco Human Rights Commission to participate with a few other trans people in an effort to educate members of the LGB/HIV Advisory Committee to the Commission about finding ways to include transgender people in protective ordinances (in those days we were either transsexuals or transvestites if we were breaking gender boundaries; LGB/HIV people were having the “wrong kind of sex,” while trans people were simply the “wrong kind of people”). It took us 2 full years of monthly meetings to get them to understand that having the “wrong” kind of sex is breaking gender boundaries, too, and that when someone beats up a cross-dresser or a transsexual person on the street, they scream, “YOU FAGGOT” the same way they do for any queer person because they think we’re all the same. And what about masculine-appearing women, and feminine-appearing men, regardless of their sexual orientation or their gender identity? Don’t they deserve protection, too? Those who object to queer people think we’re all the same! We needed to be helping each other, not tearing each other down. We needed to pass an ordinance to enable the Human Rights Commission to investigate and resolve complaints when any of us are discriminated against in employment, housing, or public accommodations. Protecting people on the basis of sexual orientation or HIV status alone was simply not enough. It’s only right that we all be treated fairly and equally under the law.

In May 1994, the Commission held a public hearing in the Supervisors’ Chamber, and trans people and our allies spoke about the nature of the discrimination we faced in San Francisco. I had bid on and won the contract to write up the report for the Commission to approve, which included educational materials, a summary of the public testimony, and enumerated findings and recommendations. The report was accepted by the Commission in September 1994,^2^ and I was asked to sit down with the City Attorneys and draft a new nondiscrimination ordinance that would include gender identity or expression in addition to sexual orientation. When the new ordinance was signed into law by Mayor Frank Jordan in December 1994 (to take effect 30 days later in 1995), I turned to the Human Rights Commission staff and said, “Guess what? Now you’re in violation of your Equal Benefits Ordinance.”^3^ They were stunned.

“What do you mean?” they asked. I didn’t know that the Equal Benefits Ordinance was not yet in effect, but I knew that the new nondiscrimination ordinance would have to be applied to any employment-related benefits somehow. I had never read any benefit policy documents available to City employees, either; nevertheless, I persisted on moral grounds, no matter how tenuous they might have seemed.

I told them, “Now you can’t discriminate against trans people in San Francisco in employment, housing, or public accommodation, including against those who work for the city, and you know you have some trans employees. They can’t access their health care benefits because your health plans contain exclusions for things like “transsexual treatments” or “sex change” that are written so broadly they give permission to nurses and doctors and administrators, as well as to insurance claims processors, to deny any care — even basic or emergency care — to people with trans histories. You need to remove those exclusions and get a plan in place that actually affirms transgender care and allows for any medically necessary treatment a transgender person needs, equivalent to the treatments available to non-trans^4^ people.” This was my Equality Framework: If you offer something for “people,” you must include all people; treating someone differently or excluding a person simply on the basis that they are transgender (or, at the time, transsexual or crossdressing) or perceived to be LGBT is prejudicial and harmful and must be prohibited.

It then took six years before we were able to implement such a plan for City and County employees, and another four years before we could get actuarial data that disproved the insurance carriers’ greatest fears: 1) This benefit will cost too much; 2) If we offer this, everyone will want to change their sex; and, 3) There aren’t enough trans people to make offering this benefit worth the effort to administer it. It turned out that none of those things were true. Trans bodies simply must be treated as equal to any other human body.

With the help of the Corporate Equality Index (CEI) from the Human Rights Campaign (hrc.org), which included transgender nondiscrimination as a foundational requirement from its inception in 2002 and continued to raise the bar with health care benefits,^5^ we have been able to secure transgender-inclusive insurance benefits at 1088 of 1271 CEI-rated companies (67% of the Fortune 500 businesses) as of 2022.^6^ By 2014, the experience in the private health insurance market, driven by the CEI, was a significant factor in the decision by the Centers for Medicare and Medicaid Services to lift the exclusion for “transsexual surgery” that had been in place for public health insurance systems since 1981.^7^

Before we got to that point, though, I was thinking about what would happen when we finally won insurance coverage for trans health in the United States: There were not enough doctors as it was already in 1995, with services available essentially through almost clandestine networks that were difficult to tap into. It was already difficult for trans people to find a doctor who would prescribe hormones, and many avoided annual exams or any kind of treatment, as much as possible, for fear of being laughed at or abused in a doctor’s office. What could we do to change that paradigm? A friend told me about a group of doctors called the Harry Benjamin International Gender Dysphoria Association (HBIGDA). This group held scientific symposia every two years, alternating between North America and Europe, at which they presented papers about their research or clinical experience working with transgender people. And they published something called the “Standards of Care.” I wondered how I could get a copy of that, but my friend told me it was just for doctors. I found out that their meeting in 1997 would be held in Vancouver, British Columbia, and I determined that I would find a way to attend.

By coincidence, I was collaborating with trans woman author, editor, and health advocate Dallas Denny on an article solicited from us for a book on bisexuality.^8^ Our chapter focused on gender identity and bisexuality, and in the course of developing the content, I learned that Dallas had a copy of the HBIGDA Standards of Care that had been published in the journal *Archives of Sexual Behavior* in 1985.^9^ Dallas had even attended one of the HBIGDA meetings (in New York in 1993), where she had been one of very few trans people present. After reading the Standards of Care myself, I knew we, trans people, had to be more involved in the establishing of criteria that both defined us and influenced our health. And if the people writing these Standards and attending HBIGDA meetings were the leading doctors designing and delivering health care and transition-related treatments for trans people, then these were the people we had to engage. If the quality of our care was ever to be improved and the number of doctors increased, then HBIGDA was the place where the knowledge base existed that would allow the medical field to expand, if only because they could help to educate other professionals.

Dallas agreed, and she encouraged me to propose a paper presentation for the 1997 meeting based on our essay that was published in 1996. My proposal was accepted, and I was also invited to speak on a panel organized by trans support providers from The Center in New York City (Lesbian and Gay Community Services Center, Inc.). I paid the registration fee and arranged to stay with a trans man in Vancouver, since the hotel was expensive. Through my Vancouver friend, I learned that local activists were planning to disrupt the HBIGDA meeting during the scheduled discussion of the revision-in-process of the Standards of Care, which, when finalized, would become the 5th version of that document. Not having any preconception of what an HBIGDA meeting would be like, I thought I’d just go with the flow.

In 1997, HBIGDA listed only 140 people in their membership directory. It was a society composed of academic researchers and clinicians in private practice who had encountered trans people and become intrigued by the questions our existence raised about the confluence of biological presumptions and social constructs, and who had come to be interested in helping us access medical care and lead fulfilling lives. Of course, there were some differences of opinion about what a “better life” would be: There were those who believed trans people were mentally ill, and that transition was to be avoided if at all possible, but they were a distinct minority. I encountered one woman who claimed to be a psychotherapist who believed that transness was a psychic disturbance akin to being inhabited by a ghost, and that she could help patients to exorcise that psychic manifestation. That woman actually commented on how real my beard looked and pulled on it to see if it was glued onto my face. A psychologist from New York suggested I should have her arrested for assault; I didn’t think it would be worth the trouble. I didn’t think she was a HBIGDA member; I think she was there trying to sell her self-published book to people in the field treating trans people. That woman and her theory have since vanished.

My presentation was well-received. The room where I delivered it was packed, and even Dallas Denny was there. She was a qualified member of HBIGDA because she worked as a psychological examiner for the State of Tennessee, but her professional work had nothing to do with trans people. Like mine, her advocacy was conducted entirely independently. Afterward she and I spoke with a number of renowned psychiatrists and psychologists who had compliments and questions for us. Most notable was Ira Pauly, a psychiatrist who had been publishing research articles on trans people since the 1960s. In the presentation, I had mentioned some terms commonly used by medical and psychological communities to refer to trans people, pointing out that many trans people find those terms “pejorative, dehumanizing, and objectifying.” Pauly asked us, “What *do* you want us to call you?” This led to several research projects, culminating in an article in *Transgender Studies Quarterly* (2018) in which we concluded that “We are fortunate in the twenty-first century that more providers and researchers are listening to our language rather than simply applying their own.”^10^

The community protest did happen as planned. During a plenary session, when everyone was present, furious banging on the auditorium doors alarmed the group of physicians on the stage, as well as the HBIGDA board members, who were in front row seats. The topic of the forthcoming revision of the Standards of Care was being presented by Dr. Stephen Levine, Chair of the Standards Committee. He was outlining the changes that brought the Standards’ structure more in line with the Diagnostic and Statistical Manual of Mental Disorders depiction of Gender Identity Disorder which had been endorsed by the American Psychiatric Association in 1994 (the last previous release of the HBIGDA Standards had been in 1990). The pounding and yelling was frightening, especially for those seated in the back, closest to the doors. As I recall, it was the Association’s president, Richard Green (no relation), who said, “It’s all right; let them in.”

The protestors swarmed in, filling every available space, cheering at first, feeling victorious, and then realizing everyone was waiting for them to quiet down so the discussion could proceed. Dr. Levine continued to present the content of the then current draft, and ultimately the most pressing demand from the protestors was that there was virtually no consideration of female-to-male transitioners in the Standards of Care. Levine replied, “Well, then, I appoint YOU”— he was pointing at me in the audience — “to review the document and advise the Committee regarding what should be added for the female-to-males.” After the meeting, I approached him and he told me I could have one week to review the manuscript and provide comments, which the committee would take under advisement. He would email me the draft.

A therapist from San Francisco who knew me through my local advocacy work there invited me to dinner with HBIGDA’s Executive Director and another psychologist who were both based at the University of Minnesota. The three of them strongly encouraged me to join the association and become active on committees to improve the way transgender people were viewed, studied, treated, and supported in society at large. I asked, “How much does it cost to join?” Dues then were, as I recall, a little less than $200 annually, which didn’t seem outrageous. “But,” the E.D. told me, “you would have to join as a supporting member. You’re not qualified to be a full member because you don’t have the proper professional or academic credentials, which means you can’t vote in elections of officers or on other Association business matters that the membership is entitled to vote on.”

“Okay, what’s the price difference, then?” I asked.

“Oh, there’s no difference,” she said.

“Really? Why should I pay the same amount and have fewer privileges?”

“That’s just what the Board has determined,” she explained.

“Well, okay. I’ll join because I do want to be involved,” I said, but I thought they could sure use a better business model.

When I got home, the draft of the Standards was in my inbox. I called a friend who could kibbitz with me and together we stayed up all night working on document, making both editorial suggestions and substantive recommendations to address trans men’s needs and treat trans men more explicitly and fairly. I emailed my finished document to eight other trans men who were known community leaders, including two attorneys, two Ph.D. psychologists, two other Ph.D. holders, one a philosophy professor and one an anthropologist, plus a Ph.D. candidate whose research was devoted to transgender health, and a trans man who had served on the HBIGDA board for many years and was a mental health provider. I told them all about the time constraints and asked if they saw anything I might have missed. The response from them was very encouraging. I incorporated a few useful suggestions and submitted the document to Dr. Levine and his committee with credit to the eight additional reviewers.

I joined HBIGDA for the 1998 year as a Supporting Member. In my welcome packet, I received my very own copy of the Standards of Care, the 1990 version.

When the Fifth Version of the Standards of Care was released in 1998, it contained none of the changes I and my colleagues had suggested. I was listed in the front matter as a consultant, as was Dallas Denny. We were the only trans people who were acknowledged. I was not pleased. I renewed my Supporting membership in 1999 and attended that year’s Scientific Symposium in London. Professor Holly (now Aaron) Devor, Anesthesiologist (and trans woman) Anne Lawrence, and Physician’s Assistant and former Board Member Jude Patton were members of the Standards Committee, and Holly had asked for a copy of the critique I had submitted in 1997. I don’t know what went on behind closed doors, but when the 6th Version of the Standards was released in 2001, many of the points I had made previously had now been addressed.

Even though I wasn’t a voting member, I still tried to make things happen by communicating directly with board members and officers who I knew were truly concerned about transgender people but weren’t always interested in instigating change or evolution. The Board seemed reluctant to let anyone know the organization existed or had a mission to increase knowledge about gender identity; they seemed content to pursue their clinical practices or their academic research or administrative roles and not make any waves. Publishing articles in academic journals seemed forthright enough for many, since that was the method they knew best, and it would help their individual careers. But I was interested in getting practical things done that would make a difference in the lives of all trans people, not the few who managed to see clinicians in the few existing programs or the few knowledgeable private practitioners, many of whom charged fees rendering themselves inaccessible to the majority of trans people in their regions. The Association itself needed to be awakened to its own power and authority, and to learn to use that power for long range impact.

As I was sitting at my desk at my job as a writer for a major financial transaction processing company one late winter day in 2001, my friend, attorney Shannon Minter, called me to ask for my help. He and attorney Jennifer Levi were working on an amicus brief on HBIGDA’s behalf in a case before the Supreme Court of Nebraska in support of JoAnn Brandon, Brandon Teena’s mother, who had sued for negligence, wrongful death, and intentional infliction of emotional distress in connection with the 1993 rape and murder of her child.^11^ The district court had found the county negligent, but reduced the damage award by 85% for the intentional torts of the convicted murders, and by 1% for the negligence of the victim, implying that by passing as a man, Brandon Teena deserved blame for making himself an understandable target. The attorneys thought that an informative brief from HBIGDA about the nature of gender dysphoria would help the court understand that Brandon was a human being who did not “deserve” to be raped and murdered. They wanted me to review the brief and to help get through to the board members who had to approve the brief that time was of the essence, and they did not have the luxury of a month or even a week to wait for approval: It was needed in just days. I dropped everything to make that happen, giving Shannon immediate feedback on the draft he sent, and I called HBIGDA’s president and insisted that the board find a better way to secure approvals for legal matters. We came to an agreement about an expedited process with a subset of board members involved. On April 20, 2001, the Supreme Court of Nebraska found for JoAnn Brandon, specifying that “the district court shall not reduce the award […] for Brandon’s predeath pain and suffering [… and…] there is no evidence to support a finding of negligence on the part of Brandon…” This was a small but important victory for trans people, demonstrating that our lives are not worth less because of our gender identity, and who we are does not justify violence against us.

At the 2001 HBIGDA Symposium that September in Galveston, Texas, my status was still that of a Supporting Member. Over 300 people attended and I was very happy to see the physicians, surgeons, and mental health clinicians beginning to respond to the trans community’s increasing visibility and demands for more respect from the medical community. Change was coming. Sure enough, in the spring of 2002, I received an unexpected email from HBIGDA’s Membership Committee Chair congratulating me on my new status as a full member of the Association. In 2003, I was elected to a four-year term on HBIGDA’s Board of Directors. I thought, “Now the real work would begin,” but I was still thwarted. The board only met once every two years, and although I was asked to lead the Advocacy and Liaison Committee when the Board met in Ghent, Belgium that year, I was not permitted to take any action without full board approval. I also requested to be a member of the Standards of Care Committee, but my request was denied. One former president told me, “We lost members because you were elected.” I replied, “Gee, that’s too bad; they’re going to miss all the fun.” The remark did sting, but I was determined to win the doubters over.

Working behind the scenes, I tried for years to gauge leading members’ interest in having HBIGDA start offering training programs. Some of the strongest leaders were dead set against it, saying there was too much liability, and we didn’t have the resources to set up and administer training programs, let alone develop and deliver them. Those who were interested were also very busy, and didn’t see how we could pull it together, especially with powerful opposition from within. I didn’t push excessively, just kept the issue alive and tried to put the idea into people’s heads that training could bring money in to support the Association, and that money would allow us to grow. Everyone agreed we needed more members, everyone agreed the work we all were doing was important, but no one wanted to make waves or more work for themselves. There were (supposedly) so few trans people in the world anyway, some thought we could go on as we had been doing indefinitely. I kept saying the trans communities were growing, there were more of us than they imagined, and they needed to keep the organization relevant. Still, the status quo seemed safer for the busy medical and mental health clinicians.

In 2005, HBIGDA’s Symposium was held in Bologna, Italy, and board members were beginning to realize that the world was changing. I renewed my request to serve on the Standards of Care Committee and was again rebuffed. However, I was heartened by that year’s election of the first transgender person as the Association’s president, law professor Dr. Stephen Whittle of the UK, who would hold the office from 2007-2009. And the board members were recognizing there was more work to do because of issues that arose, legal cases needing amici, rising demand for media interviews, and increased political activity requiring informed commentary. They decided that one board meeting every two years was not enough, and that we should have an off-year meeting, so the board met in New York City in 2006, where we discussed changing the name of the Association. I suggested we stop implying that our focus was a disease condition, and state unequivocally that our focus is transgender health, not a disorder diagnosis. We collectively hit on WPATH because we sought an acronym that had an available URL, and it had all the elements that described who we were and what we were about. Dr. George Brown and I crafted the organization’s new mission statement, which the rest of the board weighed in on, and we planned how to propose the name change to the membership for ratification. Also at that meeting, Stephen Whittle and I urged one of the officers to change his stance on denying transition-related care to incarcerated individuals, which the gentleman objected to because he felt it would encourage trans people to commit crimes in order to access healthcare. I said we needed to be advocating for insurance coverage, too, to help alleviate the kind of desperation that he was afraid of, not forcing people who had been stripped of their rights to lose access to necessary care. We came away from that meeting newly energized. It seemed to me that we were starting to turn the corner toward moving the organization forward.

In 2007, I stood for a second term on the board. I was re-elected, and was again informed privately, by the same former president, that, “We lost members because you were elected.” I replied as I had before, but this time I went away from the exchange angry since I knew our membership rolls were growing… slowly, but they were growing. The next two years were consumed with the formal name change process. There were several long-time members who had known Harry Benjamin and who revered him, who tried to organize a campaign against the name change. Benjamin had never been a member of the Association, but those who wished to honor him by naming the organization after him felt to remove his name was the ultimate insult to his reputation in the field. While I respected their feelings, I and many others believed that the health and stewardship of the organization demanded that we rebrand ourselves. The next generation of professionals didn’t know or care who Harry Benjamin was, and if we didn’t focus on health rather than disease we would be mired in the past as efforts to revise diagnostic criteria were growing and would ultimately prevail. Nothing should prevent us from honoring and memorializing Dr. Benjamin, from singing his praises and preserving his legacy, or from researching gender dysphoria and seeing how the condition would evolve, particularly as long as it remained a component of transgender health. By the end of the year, the name change had been ratified by the membership.

In January of 2008, I produced, with the support of my colleague, André Wilson, with whom I delivered numerous insurance training workshops and training webinars over the years, a four-page document entitled “WPATH Clarification on Medical Necessity of Treatment, Sex Reassignment, and Insurance Coverage in the U.S.A.” and presented it to the board for approval to publish it under WPATH’s imprint. We were at a point where such a declaration was necessary to move forward on the insurance front, and it would also be helpful in some of the legal cases LGBT legal organizations were engaged in, some of which we were also being asked to support through acting as *amicus curiae.* That June, WPATH issued that paper, its first “public policy statement,” which would be followed by many others that I wrote in response to crises or to clarify what the board agreed were ethical positions in transgender health that had not been explicitly addressed in the Standards of Care. Version 6 of the SOC had been released in 2001, and work had begun on what would be version 7. I was too busy to ask to be on the committee at that time. I was consulting with many corporations on negotiating trans health with their insurance companies and training human resources departments on making workplaces safe for trans employees. I was also working on my doctorate in Equalities Law, specializing in transgender and transsexual issues, at the Manchester Metropolitan University in England, under the rigorous supervision of Professor Whittle. I had actually enrolled there as a part-time, distance learning research student in 2003, most of my time until 2008 had been spent reading law books and reviewing cases, trying to narrow my research question. But now I needed to get that dissertation going. Originally, I had wanted to earn my Ph.D. by the time I turned 60, which would happen that November. It was clear I wouldn’t be done by then, but I couldn’t go on forever; I had barely started writing, but I couldn’t give up. And Stephen kept telling me I was writing like a journalist: I had to write like a legal scholar now. Old dogs, new tricks…

In 2009, I took a part-time job with the Center of Excellence for Transgender Health at the University of California San Francisco, in the department of Family and Community Medicine. My title was Primary Care Protocols Manager, and my job was to assemble a group of physicians as subject matter experts and create a clear, medically sound protocol to guide primary care doctors and related care providers in the basic health management of transgender patients. This was my first paid position doing trans-related work. Everything else I had done in this arena since the late 1980s had been as a volunteer or for occasional small honoraria or consulting fees (the most lucrative, but also the least frequently available). Although the pay was low compared to my corporate jobs that had nothing to do with transgender issues, I was thrilled to have the opportunity to do the work, and with a stellar group of people. My last corporate position, as Director of Technical Publications for Visa, had ended in separation early in 2007 when the company offshored my department’s function to India in preparation for an IPO later that year. I was happy to have meaningful part-time work that would allow me to dissertate as well. And then, just when I had plenty of work to do, the chairman of the Standards of Care Committee asked me to join the project as one of the nine lead authors. Finally!

In 2010, the WPATH SOC and the Medical Necessity Statement were much discussed in the ruling for O’Donnabhain^12^ in an interesting case that relied on the medical necessity of transition-related care to determine whether an individual was entitled to deduct her expenses for hormones, sex reassignment surgery, and breast augmentation from her federal income tax. Many definitions of terms were debated in the majority opinion, and the concurring and dissenting opinions, and one judge criticized WPATH for being “an advocate for transsexual persons, and not just interested in studying or treating them.”^13^ I find this castigation wholly disingenuous: Who would fault the American Cancer Society for advocating the elimination of smoking, or the American Medical Association for advocating for good nutrition in school lunches? One would think these type of positions should be taken up by these organizations, and no one dismisses these groups because they take positions to improve health for particular groups of people. WPATH’s advocacy on behalf of trans people is only criticized because the critics do not like trans people, so criticizing WPATH is a proxy for marginalizing or dehumanizing, or sometimes even inflicting harm on trans people without having to think about them as individual people.

I finished my dissertation in 2010, and defended it in 2011, earning my doctorate, which was one of the most challenging projects I had ever undertaken. The viva, as the oral defense is called in England, was brutal; defenses in the U.S. that I’ve witnessed are mild by comparison. I was so exhausted; it literally took me several days to recover. I received the notice that I had been elected president of WPATH shortly after I graduated. And, yes, the same comment about losing members welcomed me to my new role. Becoming president-elect placed me on the Executive Committee, which comprised the Association’s officers and the Executive Director, who was an employee, not an elected member of the organization. Originally, the E.D. was a member who either volunteered to do the administrative work for the Association, or who was appointed by the president or the board to do that work, processing the membership dues payments, responding to inquiries from the public, helping to organize the biennial Scientific Symposia. This work was all done in the individual’s spare time, as was all the board and committee work that members signed up for. But by the early 2000s it became clear that not only did the E.D. deserve payment for a part-time role. We also needed clerical staff support. Our membership rolls were growing (the aspersions cast toward me not withstanding) and attendance at the Symposia was increasing enough to justify these expenses. And while the full board now met annually, and was in regular email communication, the Executive Committee met weekly, and there was always plenty to discuss as we kept the organization focused on growth and sustainability. I also served on several member-led committees as a working participant: the Public Policy, Advocacy, and Liaison Committee, the By-Laws Committee, the Communications and Media Committee, the Legal Issues Committee, and the Education Committee (each of which met monthly), as well as the Standards of Care Working Group and the Editorial Board for the Association’s journal, *The International Journal of Transgenderism*, which we were ultimately able to have renamed *The International Journal of Transgender Health*. In 2013, a small group of WPATH members, including trans people and international participants, met with representatives of the World Health Organization to discuss moving “Transsexualism” out of the paraphilias section of the mental disorders chapter of the ICD and creating a new category, Gender Incongruence, in a new chapter on Conditions of Sexual Health in the forthcoming ICD-11 (which was implemented globally in 2019, but has not yet been adopted by the United States). I was also privileged to help propose and debate changes to the DSM-5 revisions of 2013, which removed the diagnosis of “Gender Identity Disorder” and replaced it with “Gender Dysphoria.” When the funding ran out for my projects at the Center of Excellence for Transgender Health at UCSF at the end of 2013, although I was not thrilled to lose the paycheck, I was relieved to be able to focus all my attention on becoming president at the end of our Symposium in Bangkok in February 2014. I knew I would be too busy presiding to do anything else for the next 2.5 years.

Visibility alone does not solve problems, but without visibility those problems don’t stand a chance of being addressed. Bringing WPATH into the 21st century, making it a truly global organization, making it responsive to trans peoples’ needs and making it an advocate for trans people’s legal, civil, and human rights has been a labor of love, as much as it’s been frequently frustrating and exhausting, even painful at times. But both the trans communities and the medical and mental health clinicians who serve us are stronger for having a professional medical association to help litigators advocate, too, while we continue to shine a direct light on the realities of trans lives. Hiding in a hall of mirrors or letting others view us through their distorted preconceptions will not make the world safe for us.

The Bangkok Symposium was our first meeting outside of Europe or North America, and it was a big financial risk, but with 525 attendees it turned out to be the largest meeting we’d held and was ultimately a great success. There, we had formally launched our Global Education Initiative, which was a project to develop education on transgender health. I had encouraged this for a long time, but I wanted to see more concrete products coming out of the group of interested members. I was convinced we needed full-time professional management to be able to implement programs and create new revenue streams that would sustain the organization through rapid growth and make it more responsive, both to our members and to the constituency of trans people to whom our members deliver healthcare.

During my presidency, more changes happened within WPATH than had happened cumulatively in the previous 37 years of the group’s existence. I did engage a medical association management firm, without which we could never have accomplished all our other achievements: I proposed dividing the world into regions as a way of increasing local focus for members, creating USPATH, EPATH, AsiaPATH, and opening the way for other regional affiliates, I reduced the membership dues, and launched the WPATH Certification Training program, which has now educated thousands of new healthcare providers. And our global membership now exceeds 4000 professionals dedicated to trans health.

We’re fighting bigger battles now, of course, and the old myths about transness and whether or not we should be allowing trans people to transition or to have health care are still with us. Visibility alone does not solve problems, but without visibility those problems don’t stand a chance of being addressed. Bringing WPATH into the 21st century, making it a truly global organization, making it responsive to trans peoples’ needs and making it an advocate for trans people’s legal, civil, and human rights has been a labor of love, as much as it’s been frequently frustrating and exhausting, even painful at times. But both the trans communities and the medical and mental health clinicians who serve us are stronger for having a professional medical association to help litigators advocate, too, while we continue to shine a direct light on the realities of trans lives. Hiding in a hall of mirrors or letting others view us through their distorted preconceptions will not make the world safe for us.

## Note

The author has no conflicts of interest to disclose.

## References

[1] The Minnesota Multiphasic Personality Inventory is a type of personality test that psychologists may use to help diagnose or evaluate personality aspects — e.g., strengths or weaknesses — that may impact proposed care or treatment. With trans people, it is sometimes used to screen for mental health conditions that might be masquerading as gender dysphoria.

[2] J. Green, “Investigation into Discrimination Against Transgendered People,” September 1994, archived with San Francisco Human Rights Commission documents, *available at* <https://wayback.archive-it.org/org-571/20220601203622/ https://sf-hrc.org/reports-research-investigations#LGBT%20and%20Intersex%20Communities> (last visited August 1, 2022).

[3] San Francisco’s Equal Benefit Ordinance (effective June 1, 1997) requires that businesses in contractual relationships with the City and County of San Francisco must offer the same benefits to employees with domestic partners and employees with spouses, and to the spouses of such employees. Although Berkeley, CA was the first city to enact such an ordinance (1985), efforts to implement one in San Francisco had been ongoing since 1982. See “Two Year Report on The San Francisco Equal Benefits Ordinance” (page 4), *available at* <https://sfgov.org/cmd/sites/default/files/Documents/Equal_Benefits_2_Year_Report_d145.pdf> (last visited August 1, 2022).

[4] Note: The term “cisgender” did not come into widespread use until after 2014.

[5] I sat on the Human Rights Campaign’s Business Council, which guided the creation and implementation of the CEI, from 2002-2007. I resigned in response to the HRC Board’s acquiescence to Rep. Barney Frank’s insistence on dropping “gender identity” from the Employment Nondiscrimination Act (ENDA), but I continued to consult with the HRC Workplace Project, developing educational materials and delivering presentations to assist corporations in demanding that their insurance companies provide policies without trans-related exclusions and encouraging supportive treatment of transgender employees as they tried to navigate their healthcare networks.

[6] See <www.reports.hrc.org/corporate-equality-index-2022> (last visited August 1, 2022).

[7] See J. Green , “Transsexual Surgery May Be Covered By Medicare,” LGBT Health 1, no. 4 (2014): 256–8, doi: 10.1089/lgbt.2014.0076. PMID: 26789853. 26789853

[8] B. A. Firestein , ed., Bisexuality: The Psychology and Politics of an Invisible Minority (Thousand Oaks: SAGE Publications, 1996).

[9] P. A. Walker , J. C. Berger , and R. Green et al., “Standards of Care: The Hormonal and Surgical Sex Reassignment of Gender Dysphoric Persons,” Archives of Sexual Behavior 14 (1985): 70–90. 10.1007/BF01541354 3977585

[10] J. Green , D. Denny , and J. Cromwell , “‘What Do You Want Us to Call You?’ Respectful Language,” TSQ: Transgender Studies Quarterly 5, no. 1 (2018): 100–110. DOI: 10.1215/23289252-4291812.

[11] *Brandon v. County of Richardson and Charles B. Laux*, 624 N. W. 2d 604 (2001) 261 Neb. 636, No. S-00-022.

[12] *O’Donnabhain v. Commissioner of Internal Revenue*, 134 T.C. 34 (2010), Docket No. 6402-06, United States Tax Court.

[13] *Id*., at 90.

